# Dicoogle, a Pacs Featuring Profiled Content Based Image Retrieval

**DOI:** 10.1371/journal.pone.0061888

**Published:** 2013-05-06

**Authors:** Frederico Valente, Carlos Costa, Augusto Silva

**Affiliations:** IEETA, Universidade de Aveiro, Aveiro, Portugal; University of Pittsburgh, United States of America

## Abstract

Content-based image retrieval (CBIR) has been heralded as a mechanism to cope with the increasingly larger volumes of information present in medical imaging repositories. However, generic, extensible CBIR frameworks that work natively with Picture Archive and Communication Systems (PACS) are scarce. In this article we propose a methodology for parametric CBIR based on similarity profiles. The architecture and implementation of a profiled CBIR system, based on query by example, atop Dicoogle, an open-source, full-fletched PACS is also presented and discussed. In this solution, CBIR profiles allow the specification of both a distance function to be applied and the feature set that must be present for that function to operate. The presented framework provides the basis for a CBIR expansion mechanism and the solution developed integrates with DICOM based PACS networks where it provides CBIR functionality in a seamless manner.

## Introduction

Radiology requires a careful interpretation of the signals present in medical images in order to provide an accurate diagnosis. Since its appearance, but particularly after the move of the imaging technologies to a digital medium, radiology has become a prevalent specialty throughout most health-care institutions. In such imaging institutions copious amounts of digital data are being created and stored. For instance, during 2006, around 50000 images were produced per day in large medical institutions [1]. This rapid increase in collected data, known as “data explosion”, is a recent phenomenon and was made possible due to both the capacity increments of the storage devices and the technological breakthroughs on imaging modalities. Nowadays, such modalities can very quickly produce images of unprecedented resolution and detail [2].

Nonetheless, the variety and quantity of the produced images can become confusing, even for trained specialists, which are reporting that information overload has decreased their productivity [1].

A promising approach to manage the “data explosion” is to allow computer based algorithms to assist in the tagging and sorting of data. The goal is to automatically extract semantic and similarity information and expose that information to a practitioner in a quick and seamless way.

The case for assisted interpretation is, however, motivated not only by time and space constrains but also by the recognition that some inter-observer variation exists due to perceptual errors or fatigue [3], [4].

Content-based Image Retrieval (CBIR) methods have shown great promise in helping practitioners sift through the large amounts of data present in medical institutions. These methods rely on the automatic extraction of content from a source image to provide the query terms for a search. In this context, content means some property extracted from the image such as color and intensity distribution, texture, shape, or high level features such as the presence of nodes or objects of interest. In practical terms, CBIR systems allow practitioners to use images from any study they are working on as query to the image database hence obtaining a set of results that, in some sense, are similar to the original image. CBIR has the potential to save a significant amount of time to practitioners, enabling them to quickly move from a source image to a set of similar ones, potentially containing diagnosis reports. These reports, when compared to the original image, may strengthen the case for the diagnosis or provide the practitioner with additional insight.

Given that radiologists often rely in second opinions in order to validate their diagnosis and increase their confidence levels, CBIR provides query mechanisms that are very close to the way a practitioner operates. Typical Picture Archive and Communication Systems (PACS), however, do not easily cater to this kind of usage. The underlying Digital Imaging and Communication in Medicine (DICOM) protocol supports only queries based on textual template matching over a limited number of fields present on the DICOM file and defined by the modality [5]. So, while the DICOM files typically store image encoding information and the settings under which a study was performed (such as radiation dosage), excepting for some DICOM Structured Reports, not much information of semantic value to a practitioner’s diagnosis can be found on those files. Furthermore, the DICOM fields which are actually indexed and made available to query depend on the particular PACS provider and are typically limited to the patient name, modality and UIDs further hampering the usefulness of the protocol query mechanisms.

In this article we propose a methodology, and discuss a working implementation, for a profile-based CBIR system aimed at PACS networks. Relying in the concept of metric spaces, an approach validated by previous works in the area [6] [7], we define similarity as a proper distance (a metric) over a subset of a feature space. We detail how we have expanded an open-source PACS, Dicoogle, to support the data mining and indexing mechanisms to cope with automatic extraction of image content information and support query-by-example on medical images. Via support for DICOM QR (Query/Retrieve) mechanisms our solution can be seamlessly integrated with functional PACS networks and provide drop in CBIR functionality. The focus of this article is placed on the methodology, the architecture and implementation of the tool and respective PACS integration. Further analysis is being performed in order to access the clinical validity of the similarity functions employed.

In the next sections we provide an overview on both PACS and CBIR technologies. Section IV provides a brief overview of the related works in the area. In section V we expose Dicoogle’s CBIR software architecture. The section thereafter details the methodology employed, the features, and metrics currently used. Subsequently we provide our results, point out some directions for future development and research and present our conclusions.

### Picture Archive and Communication Systems

**Figure 1:**
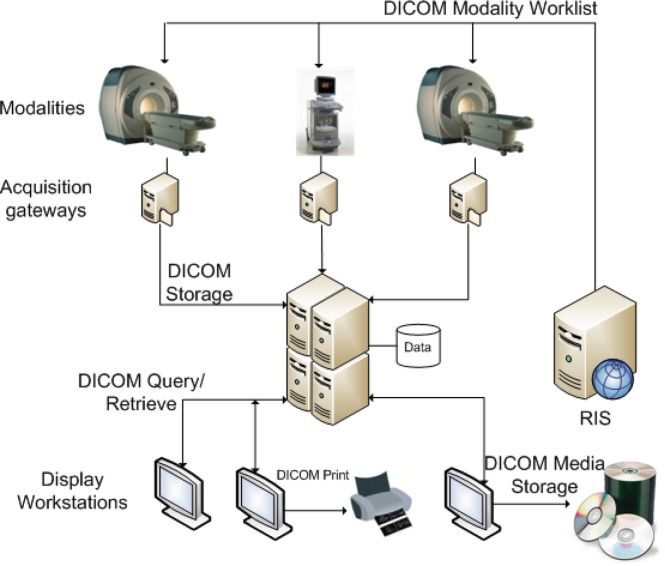
PACS overview.

Medical imaging has evolved to become a very valuable tool in both health-care and research institutions. It is now considered as a key factor in the process of providing quality diagnoses and supporting practitioners’ decision-making [8], [9]. While in its early years radiology required some form of physical support for the images produced, nowadays the process of image creation, storage and consultation is mostly digital. The move towards digital radiology gave rise to a set of challenges leading to several implementations of what are commonly designated by the umbrella term of PACS. The PACS concept is the embodiment of distinct hardware and software technologies comprising medical image and data acquisition equipment, subsequent storage equipment, and display subsystems, all of which are integrated by digital networks and end-user software [8] (see figure 1). Such systems are designed to cope with the high storage needs and transmission requirements of medical institutions. Besides radiology, several other clinical areas have been adopting PACS in their daily routines, such as cardiology [10], dentistry [11], and pathology [12].

PACS rely heavily on a set of standard definitions and communication protocols, DICOM [13]. The DICOM protocol was, by itself, a major contribution to the exchange of structured medical imaging data. It is estimated that over one billion diagnostic imaging procedures will be performed in the United States during 2014, comprising approximately 100 petabytes of volume data [14]. Given the increasingly higher demands placed over PACS solutions and the expected data growth, research in the area of PACS is very active. New technologies are being actively explored to help with data storage and management. Some directions in which new PACS systems are being investigated include distributed and heterogeneous computing grids [15], [16], Cloud Computing [17], Peer-to-Peer networks [18], and knowledge extraction using indexing engines [19]. Our contribution in Dicoogle focus on bringing more advanced and seamless mechanisms for data searching.

#### Dicoogle PACS

**Figure 2:**
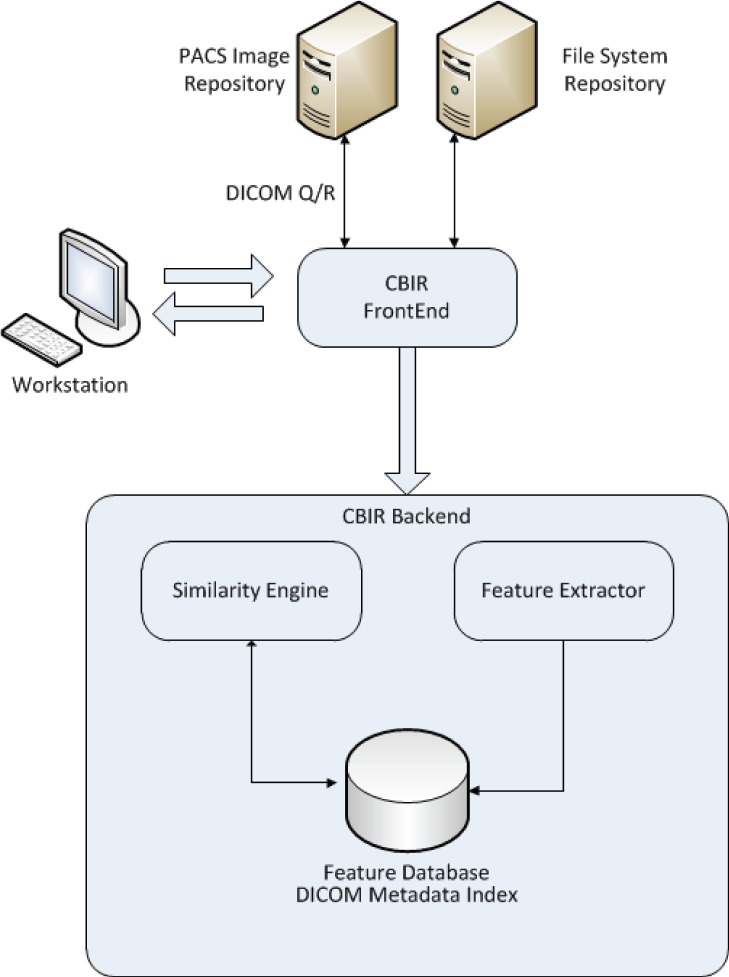
Dicoogle CBIR components.

Dicoogle (http://www.dicoogle.com) is an open source PACS that distinguishes itself from other PACS by making use of peer-to-peer technologies and document-based indexing techniques (built atop Lucene search engine library), rather than the more traditional approach of using relational databases [18]. Dicoogle’s DICOM functionality allows the application to be used as a stand-alone PACS or to access an external PACS network and index its data with minimum configuration and close to no disruption of both an institution’s workflow and network (see figure 2). This proves useful when performing data-mining operations as done in [20]. Dicoogle also features an extensible plugin-based architecture, which we leverage to provide CBIR functionality. Since very few PACS natively support CBIR, an external deployment of Dicoogle can provide drop in CBIR functionality into an institution’s PACS.

### CBIR

Content Based Image Retrieval can be defined as the set of technologies that help to organize, search and retrieve images from digital picture repositories according to their visual content. This is a broad scope definition of which several distinct approaches, ranging from similarity matching techniques to interpretation engines and image tagging, fall under [21]. Ideally, however, CBIR engines should extract data directly from an image’s content with little to no intervention from the user, in this case radiologists. Due to the complexity of the task and ambiguities arising from segmentation and image analysis this is not always possible or even, in some cases, desirable. A fully automated approach is, however, the one taken in Dicoogle. This is because one of the goals is to allow image indexing and seamless integration with external PACS which may comprise a very large number of images.

A CBIR architecture can be streamlined into a set of distinct components, the variations within them are what distinguish amongst various CBIR strategies and implementations:

- Data sources - Components responsible for image acquisition.
- Feature extraction module - Extracts features from images and creates a representation suitable to the feature database.
- Feature or image database - Stores, and possibly indexes the features for fast searches.
- Similarity engine - Is the component in charge of defining the similarity between images and performing comparisons between images or features.

Conceptually, Dicoogle CBIR plugin also follows that architecture (see figure 2).

#### Image features

The most direct approach to compare images is to match pixel data directly. This approach however is generally not feasible as it may not be clear which pixels from one image correspond to which pixels in the other image. Direct pixel comparison is overly sensible and breaks down when images have been taken under different lighting conditions with distinct resolutions. Furthermore, besides being a very slow operation, there is the problem of how to properly index the image in such a way as not to have to analyze the entire dataset for every query made.

Since Dicoogle’s goal is to cope with large imaging datasets where most imaging information is either redundant or irrelevant, the analysis is preceded by a feature extraction stage that provides a reduced representation of the original data in the form of a set of features. That said, a feature is simply a relevant piece of information, a synonym for an input variable or attribute of an image, such as lines, shapes or textures [22], smaller in size than the original data. Using a feature based approach helps reduce the size of the data that must be stored and provides superior generalization capabilities and much better performance than direct pixel-to-pixel comparison. Of interest is that some features, being high-level representations of an image, can embody a particular concept and allow similarity models to become both more specific and accurate while helping bridging the semantic gap [23].

#### Metrics for image similarity

How to define measures of similarity between content, or features, and how to assess the results is an ongoing challenge in the area [3] and a topic garnering the interest of a large number of researchers.

Implicitly, one person has a clear notion of whether any two objects or images are similar. A trained opinion may have a different notion of whether two images are similar. Nonetheless, even for trained eyes, a certain amount of subjectivity is at work and it influences diagnoses in radiology. Misdiagnoses by radiologists due to non-medical reasons are reported to be in the range of 2% to 4% [4], [24].

In order to provide a user with mechanisms to retrieve objects similar to a query image, we must first consider what similarity is.

In this work, as in [6] [25] we follow the metric space approach to similarity. Hence, in accordance to [7], we define similarity as a distance function, 
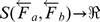
, over two elements of the feature space. Technically we use a dissimilarity function, that is, a function that returns 0 if, and only if, 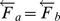, and increases in value the more distinct both images are.

A valid similarity measure, being a distance, must therefore conform to the metric postulates.

So, if 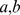 are elements of the feature space 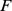, and 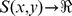 is our similarity function, then:

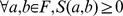
 non-negativity

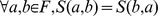
 symmetry

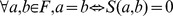
 identity

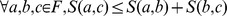
 triangle inequality

The similarity function can then be used to establish an ordering through a set of elements 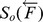 relative to the the original query object.

Furthermore, relying on the triangular inequality, a property of all proper distance functions, a wide range of metric indexing mechanisms can be leveraged to provide increased performance [7] [26] [27].

In order to provide medically relevant queries via a similarity function a tight relation between the feature set and the distance function must be established. For instance, a feature set comprising edges and segmentations can be more useful to establish shape similarity than an intensity histogram and entropy. Discriminative features with semantical meaning greatly simplify the metric. In general, the more semantic is embedded in a feature the simpler we can make the metric. There is then a strong dependency between the similarity function and the features extracted and subsequently stored, and successful image retrieval depends on both aspects. Furthermore, the discriminative capabilities of a feature are very dependent on the context in which they are applied. For instance, masses and nodes are unlikely to have any meaning unless we are handling mammograms. This means that both the extracted features and similarity functions employed can become very modality dependent. Features and functions that efficiently represent similarity within the scope of a modality can be entirely not applicable to any other modalities.

### Related Work

Content based retrieval systems are currently being deployed for a variety of purposes, in the form of facial or character recognition, rhythm detection and comparison, and some forms of template matching and nearest-neighbor classification. There is also a big push to bring this set of technologies into the medical arena, however, the specificities of the area make this a very challenging task. A very good survey of some state-of-the-art approaches to CBIR in radiology can be found in [3]. Typically, the medical CBIR systems presented in [3] are very focused in a particular modality and methodology. Moreover, most presented systems are research projects which operate directly with a set of images and do not concern themselves with integration with neither the DICOM protocol or a PACS. In [28], however, a medical CBIR system is presented which integrates itself with the PACS and the Radiology Information System (RIS) from the University Hospital in Aachen, Germany. This solution, however, lacks the profile support and drop in functionality our system provides.

A wavelet based approach is also presented by [29]. This system indexes images in a generic fashion, without using domain-specific features, employing instead a signature built from an image’s wavelet transform. A non-medical, but related CBIR project is Lire. This project, like Dicoogle’s, also leverages Lucene’s search engine to index imaging features and provide a framework to perform CBIR [30]. However, it only supports sequential searches through the dataset and its scope is very generic.

## Methodology

In this section we detail the compromises and strategies followed to bring CBIR into Dicoogle. These choices were partly defined by the fact that we already have a working platform, powered by Java and using Lucene as its data backend, with a Software Development Kit (SDK) that allows for extension of functionality.

A set of considerations have guided our approach to bring CBIR to Dicoogle. Firstly, we desire to keep providing the user with a turn-key solution, one that works out of the box without the need to install third-party components or install and configure external databases. A second consideration is that the should work with as many medical modalities as possible, with minimum input from the user. Our final consideration relates to the expandability and orthogonality of the tool and respective code. It must be simple to expand Dicoogle with new metrics and features and those must make use of the already present indexing mechanisms.

The metric approach to similarity was employed since it is a very general mathematical framework that still enables the creation of indexes over the data for faster searching mechanisms. Metric indexes have also been reported to be more robust against the “curse of dimensionality” [7].

### System Architecture

**Figure 3:**
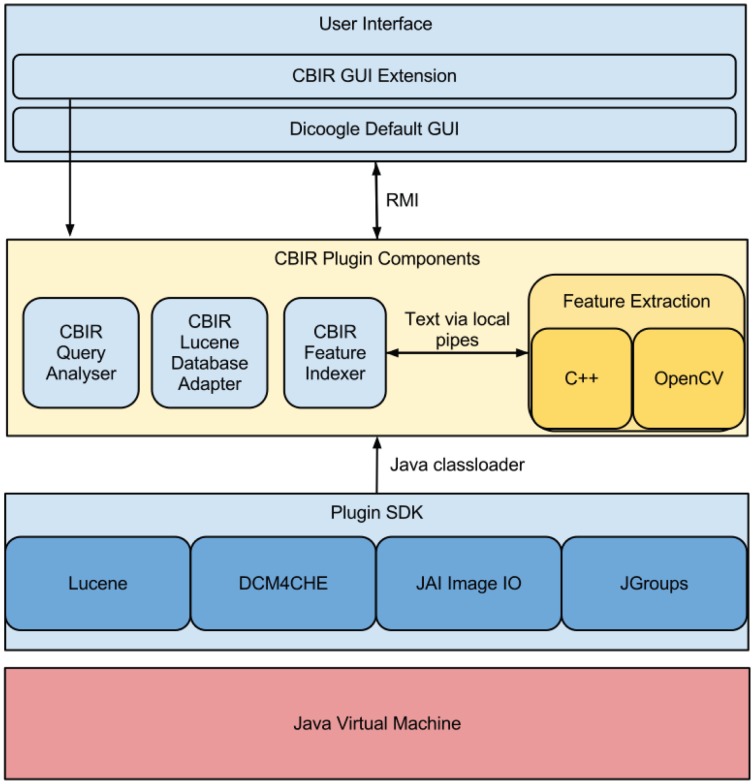
Dicoogle’s Components.

The Dicoogle application is a multi-platform, plugin-based open source project developed in Java and tested in three major operative systems (Linux, MacOS X and Windows). In figure 3 an overview of the main components and tools are shown. The application graphical interface is separated from the core application through the usage of Remote Method Invocation (RMI). Doing so makes it possible to run the core components (indexing, storage and service provision) on one machine, and the graphical interface on distinct machines while supporting multiple users.

Dicoogle’s DICOM functionality (data extraction and service provision) is built on top of the DCM4CHE2 library [31]. As for the indexing mechanisms, they make use of Lucene, a full featured text search engine with high performance [32].

The newly implemented CBIR functionality rests atop the plugin system and can be loaded automatically or on demand. Due to performance constrains it makes use of a feature extraction program written in C++. The feature extraction program leverages OpenCV, an image analysis library commonly used in robotics and computer vision projects, to provide the basis for the image analysis and feature extraction algorithms. The feature extraction program is compiled for the three major operative systems and architectures hence allowing Dicoogle to remain by and large a multi-platform application.

### Profile Guided Content Matching

There are several, very distinct, approaches to perform similarity retrieval or computer aided diagnosis. Approaches range from the usage of Support Vector Machines (SVM) [33], clustering of micro-calcifications in mammograms [34], image patches and bag-of-features [35] or wavelets [29]. These methods are focused on different aspects of an image, often only working with a specific modality, and tend to give prominence to a certain aspect of an image in detriment of others. As mentioned, when employing multiple features to sort by similarity, that similarity must be defined. In the context of mammograms there is a tendency to focus on micro-calcifications to provide the relevant similarity rather, than, for instance, tissue type or size of breast.

Furthermore, image analysis is a very volatile field, where new approaches are constantly tried and old methods improved. To cope with the dynamic requirements imposed by the need to refine both the similarity models and features extracted, we have decided to follow a general and expansible approach. Therefore we have separated the similarity metric from the feature extraction and indexing processes and provided the user with the concept of “CBIR profiles”.

A profile contains information on the metric to be used and which features are required to successfully apply it. It also contains information on how to extract candidate points, thereby limiting the search space, and on which modalities its use is meaningful. Due to the dynamic capabilities of our CBIR engine and Lucene’s database we can make the process of defining features and similarity functions dynamic, with no changes required to the core engine which will store and index those features.

Using profiles, our CBIR engine allows a practitioner to specify what is of interest to him and fine tune the query if required. In order to simplify the interface with the user, Dicoogle analyzes the modality of a DICOM file and it can automatically select an adequate profile.

Currently, Dicoogle supports two profiles. A general purpose CBIR profile, using exclusively global image features that are always indexed and can be used to compare between any type of image, and a profile specific for mammograms.

### Features Extracted

When using a profile based methodology it is a requirement to know in advance if there is no mismatch between the extracted features and the features required by the profile. We have divided features into feature sets. General image features, which can be extracted from any type of image and can therefore be applied to queries over any category of image, and modality specific features, which being more specific and encoding domain-specific knowledge apply only to certain modalities. This enables us to perform CBIR in a modality independent fashion if we wish, albeit with worst results, or use special purpose algorithms and classifiers to extract and index particular features.

Currently, Dicoogle’s general CBIR profile makes use of the following features:

- Intensity histogram
- Edge histogram
- Entropy
- Segmentations and respective area and center of mass
- Image momentums

As mentioned, DICOM files generally contain their modality as part of the file meta-data. Using that information, feature extraction algorithms can take context into account and embed expert knowledge or a-priori conceptions about the semantics of the modality and image into the feature extraction and comparison process. When a mammogram is found, instead of extracting only general image features, we look for micro-calcifications as well, likewise for distinct modalities. These features are then stored until a profiled similarity function makes use of them.

An interesting use case arises when these specific high level features have semantic value, such as micro-calcifications. In such cases we can expose that information to the underlying database (Lucene) and allow for semantic textual queries directly from the query language.

When a mammogram is detected, besides the general image features the following features are extracted as well:

- Calcification candidates
- Breast segmentation, and respective area, center of mass and average edge angle to center of mass
- Texture Descriptors

### Metrics and Similarity

Not unlike features, the metrics for similarity can vary depending on the profile selected. Although currently we lack expressiveness on the profile file to express any arbitrary function, the following metrics are supported in profile creation:

- Euclidean Distance
- Weighted Quadratic Distance [36]
- Earth Mover’s Distance [37]
- Bhattacharyya distance [38]

The distance measures can be parametrized adjusting weights on a predefined number of parameters on the profile. These functions are then applied to the subset of the stored features specified on the CBIR profile.

### Feature Indexation

At the application’s core, Lucene search engine is used to create and indexes for fast data retrieval. Dicoogle makes use of two distinct indexes, one to index the meta-data embedded in the DICOM file, useful to query for information such as radiation dosage, modality or patient name among other information. The second index is used to store the image features.

**Figure 4:**
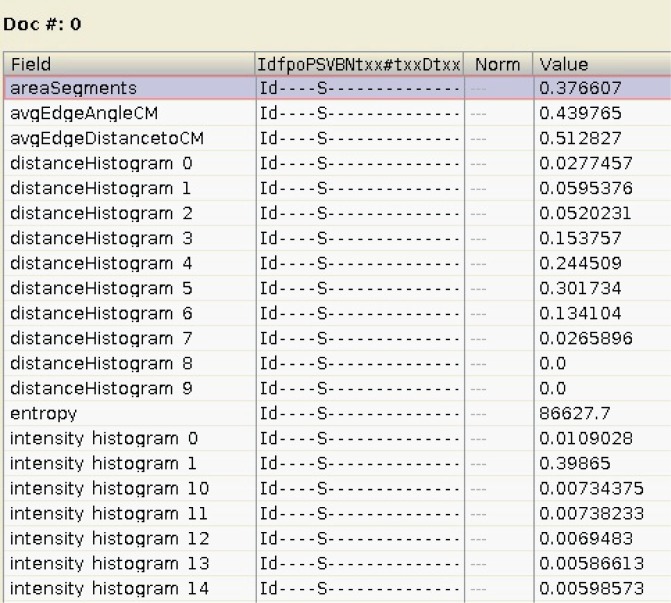
Overview of a Lucene’s document of features.

Lucene operates on a document-based paradigm [32]. This means that, for each indexed DICOM image, a document is created. Documents contain a number of fields which are used to store the features in a textual format, a limitation imposed by Lucene, but which is still apt for querying. The created Lucene CBIR documents are then a semi-structured collection of features with a reference to the original DICOM file. Although not strictly needed, multi-dimensional features (either vectorial or histogram-based) are split into several distinct document fields indexing each of them separately. This allows us to sift and discriminate on single dimensions of the feature and cull the search space more effectively, whether by applying heuristics or focusing on only the relevant elements depending on the candidate selection algorithm. To query those features a common naming convention was employed where the prefix is defined by the global feature name (such as edgeHistogram) and a numeric suffix is appended according their respective dimensional index (see figure 4).

Given that Lucene is a full-text search engine and not a conventional database, numeric fields are converted to lexicographic sortable string representations and the search mechanism is based on a trie algorithm as presented in [39].

Furthermore, Lucene’s data aggregation mechanisms support dynamic insertion and querying of new data fields. In practical terms, this allows Dicoogle to evolve its feature extraction algorithms and features employed without altering the indexing and query mechanisms.

### Query by Example in Dicoogle

**Figure 5:**
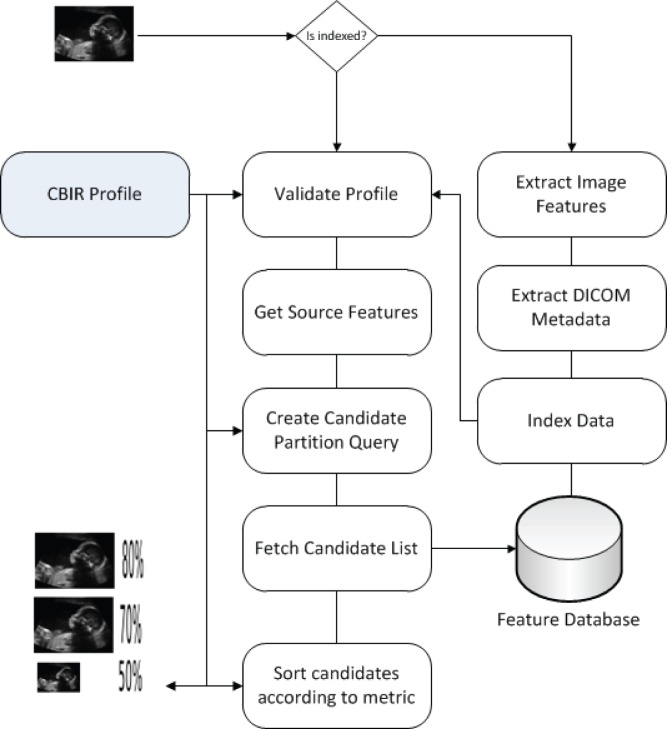
Dataflow diagram for Dicoogle’s query by example functionality.

Dicoogle’s CBIR functionality is exposed to the user via query-by-example. A query by example is initiated from the Graphical User Interface (GUI) by selecting an image and a profile. For an overview of the process consult figure 5. Typically a profile is selected automatically, if an adequate one exists, for the modality in question. Otherwise, the general image CBIR profile is automatically selected. The system performs a check to verify if the source image already had its features indexed and extracts them if not.

**Figure 6:**
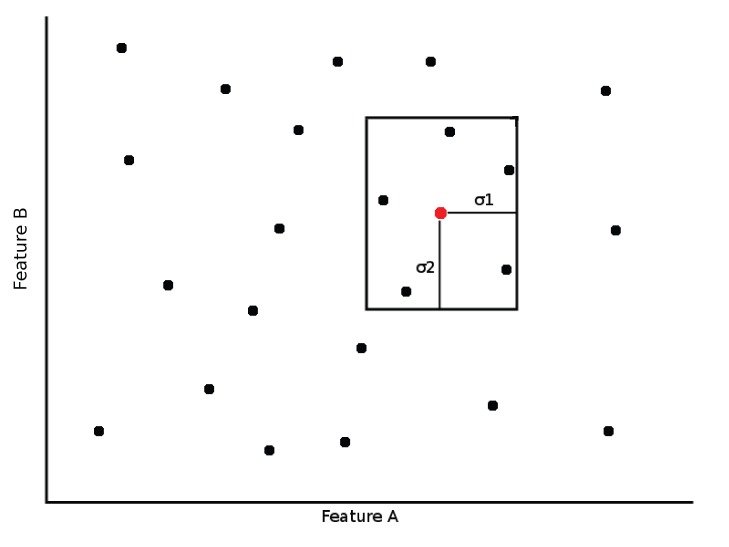
Feature space division using the query values (red point) as source for a bounding box.

**Figure 7:**
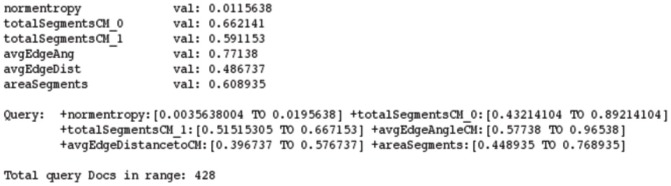
Feature values for a sample image and respective query.

Taking into account the original features, a n-dimensional bounding box centered on the source is created as exemplified in figure 6. This box is allowed to encompass the entire feature space in order to provide full sequential searches as well. These bounding boxes are calculated taking into account the standard deviation of the samples or based on a range specified by the profile. Documents in the feature space contained inside the bounding box are added to the candidate list. The candidate list is populated by retrieving Lucene’s documents using a specifically crafted query. This query is created by performing a binary conjunction over the profile’s fields of relevance. For instance, a simple shape metric using a small subset of features yields a feature query as shown in figure 7. The final step is to sort the candidate list according to the metric specified on the CBIR profile. This list contains the DICOM Unique Identifiers (UID) which can then used to push files via the standard DICOM C-Move to a practitioner’s workstation or retrieve the files directly for viewing in the GUI, which, since we use RMI, can be either local or remote.

## Results and Discussion

The discussion and results presented focus only on the technical merits of the solution with no claims being made relative to the clinical aspect. Studies are currently being conducted in order to validate the approach in clinical terms.

The presented results were taken measuring the values five times and averaging them. The machine where the tests were performed is a Linux box (kernel 2.6.43) with a dual-core Intel Xeon (cpu family 15, model 2) with hyperthreading enabled and 3 Gb of RAM. The Java virtual machine executing the application is Oracle’s implementation, version 1.7.0. The feature extraction applications are built using g++4.6.3 using OpenCV 2.3.1.

The dataset used throughout the tests is provided by the Breast Cancer Digital Repository (http://bcdr.inegi.up.pt/). This repository contains mammograms of clinical cases annotated by expert radiologists. These annotations can become very useful when validating the clinical side of the solution. The provided images were not in DICOM native format, however, and were therefore converted using a custom tool.

**Figure 8:**
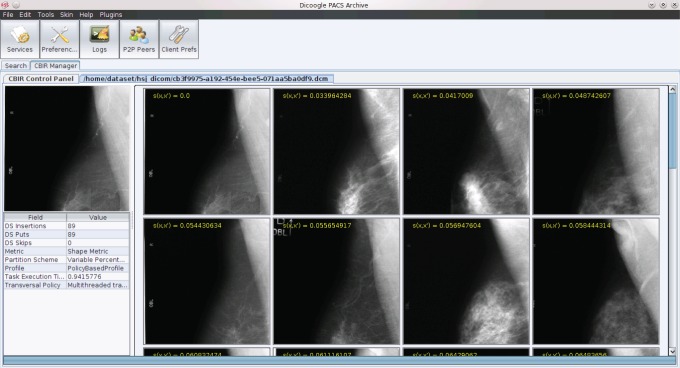
Dicoogle’s CBIR results.

A screenshot of Dicoogle after performing a query by example using a shape-based profile can be consulted on figure 8. The image on the left corresponds to the source DICOM image, used to initiate the query, while on the right canvas the returned images are displayed together with their distance to the source. For exemplifying purposes we have used a shape-based profile.

**Figure 9:**
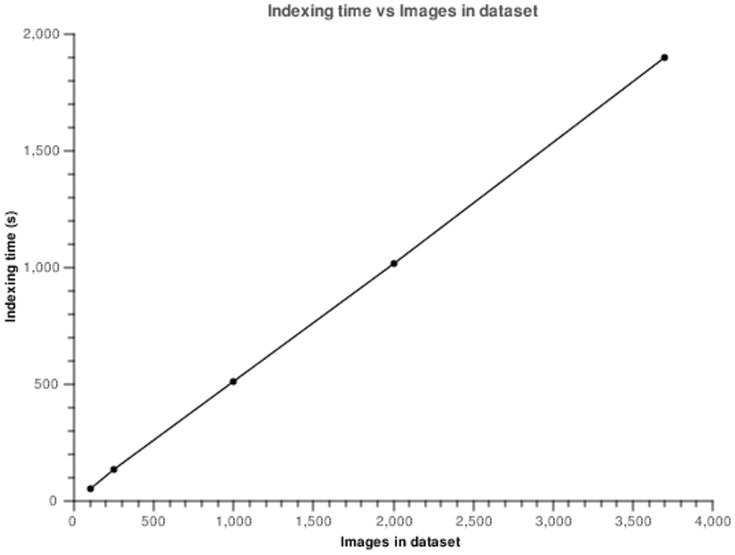
Plot of index time vs dataset size.

**Figure 10:**
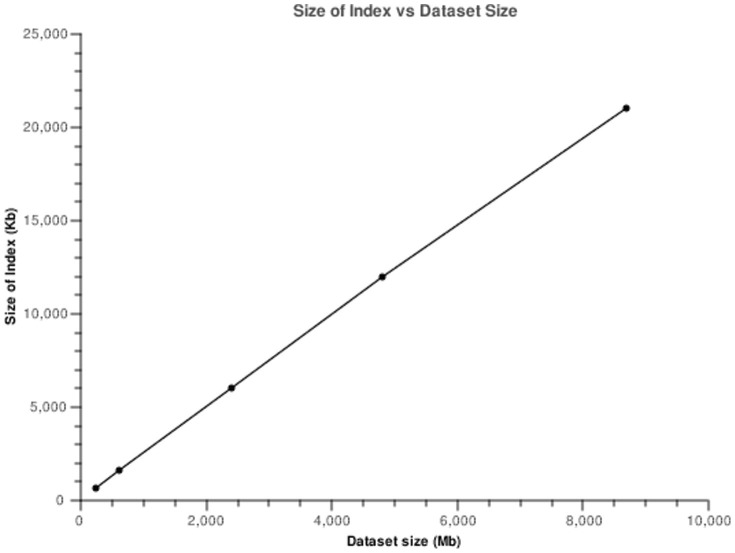
Plot of index size vs dataset size.

Typically, the feature extraction process takes an average of 2.1 seconds with some variation according to the complexity of the image. The values oscillate between 0.6 seconds to 6 seconds. Given that the feature extraction process has no dependencies with other operations it is executed in a multi-threaded fashion roughly decreasing indexing time by the number of physical cores. The values for the index build up time are presented in figure 9, and in figure 10 we show the evolution in size of the feature database, backed by Lucene.

From the analysis of figure 10 we can observe that indexing and storing the image features is an operation that scales linearly with the size of the dataset. For the full dataset, composed by 3712 images occupying 8.8 Gb of storage space the indexes take up a very small percentage of space, 21 Mb. The addition of new features should increase the index size in a linear manner, making this a scalable approach.

A query response time depends on which metric the profile defines and on which candidate selection algorithm we apply. The exemplifying shape queries typically take between under 2 to 10 seconds for a sequential transversal of the entire set. When culling the candidates using ranges defined by the profile and source element, we can cut down the response type to sub-second times, but depending on the aggressiveness of the pre-selection, the quality of the results can decrease. We also observed that parallelizing the process of candidate transversal, while performing the similarity sorting, does not speed up the application significantly, suggesting a bottleneck in data retrieval.

### Future Work

In this section we show some of the shortcomings of this approach and point some directions as to how this tool can improve. The most obvious direction in which Dicoogle’s CBIR can be expanded is to add the support for extra CBIR profiles and features. Being an open source project, with a decoupled, plugin-based architecture the task of integrating new feature extraction mechanisms and metrics should prove to be simple to implement by an interested party.

Regarding the n-dimensional bounding box, this is a crude mechanism for performing candidate selection. Valid candidates may lie on the outside of the bounding box for which the distance similarity function yields lower values than some of the inside candidates. On the other hand, if we pursue more aggressive culling strategies, too few candidates may be selected for analysis. That is, no assumptions can be made as to the number of elements inside the bounding box. A proper metric index such as M-trees, VP-trees [26], indexes based on clustering or Locality Sensitive Hashing [27] should be employed. This will have the potential advantages of providing both more accurate and faster retrieve times. Furthermore, having indexes based on the metric, rather than an index per feature dimension, means less indexes, more opportunities to compress data and may allow for a large set of features to be explored while keeping real-time performance.

Relevance feedback is an important mechanism for interaction with practitioners and to provide more accurate retrievals which we currently have not yet implemented. Its usage has already been applied to CBIR systems with favorable results [40] [41].

A most promising direction to look at is to conjugate CBIR queries with information provided in the DICOM file meta-data, which we already index. In the same spirit we wish to adjust CBIR profiles to semantic concepts such as tissue type, size or the presence of micro-calcifications so that we may enrich Dicoogle’s query language with high-level semantics.

Finally it are the researchers’ beliefs that directly extending the DICOM protocol to support profile-based CBIR and feature storage may help to bring this type of mechanism to a wider audience while facilitating data sharing within an institution and allowing for faster development and deployment of CAD applications.

### Conclusion

We have presented a non-intrusive PACS with CBIR capabilities, Dicoogle. This tool can act as a stand alone PACS providing DICOM services to small to medium institutions or as a useful component left outside the main network, communicating using DICOM and providing advanced query mechanisms and CBIR to practitioners or students. The CBIR functionality is provided according to designed profiles that may focus on a specific modality or only generic features in a modality independent way. The system is shown to be fast for the workload it was tested with and is presumed scalable. We have also pointed out some shortcomings of this approach and left directions on how this tool can be improved. It is our believe that extending this approach to work directly with DICOM in a standard way, negotiating CBIR profiles between workstations and PACS can open CBIR to a wider audience.
